# “When I Can Make Them Smile”: Cash Transfers and the Joys of Mothering in the Context of Poverty

**DOI:** 10.1002/pam.70103

**Published:** 2026-03-24

**Authors:** Sarah Halpern-Meekin, Jill Hoiting, Ruby Mendenhall, Michelle Spiegel

**Affiliations:** 1 School of Human Ecology & La Follette School of Public Affairs, University of Wisconsin-Madison, Madison, Wisconsin, USA; 2 Sandra Rosenbaum School of Social Work, University of Wisconsin-Madison, Madison, Wisconsin, USA; 3 Department of Sociology and African American Studies, Carle Illinois College of Medicine, University of Illinois, Champaign, Illinois, USA; 4 Independent researcher

## Abstract

Poverty researchers focus on whether interventions relieve material hardships and the stresses of financial insecurity. Although these are essential outcomes, a narrow focus on them can limit the evaluation of policy effectiveness. Further, the predominant focus in the field on individual outcomes may miss how policy matters for family relationships. The present study takes a relational perspective and attends to positive policy outcomes such as joy. It uses the case of unconditional cash transfers to show the joys of mothering and the role of such policy interventions in these experiences. From the Baby’s First Years study, we analyze interviews with 80 women, who were living below the poverty line at their child’s birth; 65% identified as Black. We highlight how mothers use the cash transfer money to create opportunities for joy and bonding in their mutually rewarding relationships with their children. We show how financial resources serve purposes beyond meeting material needs and as human-capital investments. Financial resources are also conduits for relationship building and family flourishing, including through joy. To holistically assess policy impacts requires a comprehensive picture that allows scholars and policymakers to understand how government programs can help families not just survive but thrive.

Poverty researchers mostly focus on what parents with low incomes lack materially and how efficacious policies are at addressing these needs. These studies emphasize parents’ stresses as they care for their children and the negative repercussions for children when their mothers experience vulnerabilities such as poverty (e.g., Burton 1992; McLoyd 1990; Norcross et al. 2020; Sharlin and Shamai 2000). Although it is incredibly important to address social problems when considering policy change, such an approach risks ignoring the complexity and reality of mothers’ daily experiences (for discussion of challenges, see Arditti et al. 2010; Arendell 2000; Mendenhall et al. 2020). When policy responses are spurred by a stress-focused perspective, the goal is to alleviate suffering—eliminating negative outcomes—rather than also promoting positive outcomes. A multifaceted approach, like the one for which the present study argues, substantially expands the possible goals of policy and intervention programs and the metrics against which we can evaluate their success. Therefore, when assessing the efficacy of policy interventions, we must also include a focus on mothers’ and families’ strengths, such as positive emotional and relational experiences, to gain a more holistic understanding of how interventions work and for whom (Pattillo 2021).

The mainstream policy and poverty studies fields are committed to evidence-based policymaking (Haskins 2017), but evidencebased policymaking is only as effective as the evidence we seek. If we systematically do not ask certain questions or take holistic perspectives, we will not know how policy affects emotional and relational realms—which are relevant for flourishing—nor how those realms may affect more traditional policy outcomes like human capital development (Elazar 1984; Robert and Zeckhauser 2011). In the present study, we move beyond the focus on life satisfaction and related measures that have received primary attention in previous work considering subjective well-being in policy analysis (e.g., Maccagnan et al. 2019; Meynhardt et al. 2024; Pacek et al. 2019; Takahashi et al. 2021). We draw on the concept of joy, a distinct component of well-being that is fundamentally emotional and relational (Watkins et al. 2018), as an example of the type of positive emotional and relational outcome that could also receive attention in policy research.

In the present study, we assert the importance of policy analysts attending to (1) positive, eudemonic outcomes, including joy, and (2) the way policy interventions work through shaping relationships, rather than just impacting individuals. Even in areas where positive outcomes may be more likely to be considered, such as child care or early-life experiences, the perspective is often individualistic (see, e.g., Currie and Rossin-Slater 2015; Herbst 2023). A few studies have shown vital policy insights that can come from including more holistic perspectives that include consideration of positive and relational experiences (see, e.g., Barnes et al. 2023; Barnes and Nolan 2019; Halpern-Meekin 2019; Randles 2014; Small 2006). Guided by the insights of this previous holistic work, we use the case of monthly unconditional cash transfers to learn how mothers discuss the relational and emotional meaning of their time with and use of resources on their children. What do we learn if we center relationality and attend to the presence of joy? What if we move beyond the more typical investments-in-human-capital perspective in seeking to understand an income-based intervention?

Using the case of cash transfers to ask these questions aligns with current poverty interventions. Recent years have seen growth in unconditional cash transfer programs and basic income interventions—such as the Magnolia Mother’s Trust, the Stockton Economic Empowerment Demonstration, and a proliferation of sites in the Mayors for a Guaranteed Income network—alongside burgeoning policy interest in a monthly cash benefit for families in the United States (e.g., the briefly expanded Child Tax Credit in 2021). Although some previous research on cash transfers has considered their impact on subjective well-being (Gennetian et al. 2024; Hanna et al. 2025; Kilburn et al. 2018; McGuire et al. 2022; Natali et al. 2018), these studies have only been done in developing country contexts and using a quantitative approach, which does not tell us what participants themselves see as meaningful emotional experiences. In addition, these conceptions of subjective well-being have often focused on summative statements of individual well-being (e.g., happiness, life satisfaction). Such conceptions of individual well-being are distinct from eudemonic well-being—like joy—as eudemonia has to do with a sense of meaning, purpose, autonomy, and connection in life, and it plays a distinct functional role in well-being from happiness and satisfaction (for discussion, see Clark et al. 2008). Therefore, the present study encourages researchers interested in assessing subjective well-being to consider a wider array of outcomes.

Further, when scholars have considered relational outcomes in their research on cash transfers, the focus has been restricted to intimate partner violence and romantic relationship quality (Baranov et al. 2021; Pilkauskas et al. 2023). The present study addresses these scope limitations by drawing on qualitative data from a cash transfer study in the United States to understand what participants themselves find joyful in the context of their relationships with their children, learning how they use financial resources to cultivate relational joys. To do so, we follow Emmons’ conception of joy: “first, as a singular event or transient state, punctuating our quotidian condition with hints of larger purposes; second, as a more durable condition or disposition that humans can pursue” (2020, 1; also see Bonilla-Silva 2019).

Taking a relational and positive emotional perspective also resonates with racial and economic justice. Work by Zora Neale Hurston (1990) and more recent scholars highlight the radical importance of centering joy, not just suffering, in stories about and research with marginalized groups. The goal is to recognize but not define groups by their experiences with oppression and to represent them in the fullness of their humanity (for work on joy, see Baker 2021; Curington 2024; Lu and Steele 2019; Pattillo 2021; Stewart 2021). This means not just looking at marginalized groups’ resilience after negative experiences (McLoyd 1990), but understanding how they cultivate positive experiences that allow them to flourish, even during difficult times (e.g., in conditions of racism, sexism, poverty, etc.). As curator Elaine Nichols (2024) put it, “Black Joy is finding the positive nourishment within self and others that is a safe and healing place. … It is a well-spring of power that is uplifting and life-affirming.”

We examine joy in the context of parenting. Often, the intersectional nature of the parenting experiences of women of color has been ignored (for exceptions, see, e.g., Dow 2015, 2019). Economic and family spheres were traditionally experienced as separate or gender-specialized in White, middle-class families. These distinctions are reflected in research divisions, which often examine economic and relational outcomes separately. However, economic and family spheres have been more intertwined in the lives of women who are marginalized (Collins 1987, 1994; Dow 2016); our approach centers this intersection.

In the present study, we examine the joys of parenthood as described by mothers who have low incomes and are predominantly women of color. We do so using in-depth, semi-structured interviews because, as Pugh (2013, 42) notes, “interpretive interviewing allows researchers access to an emotional landscape that brings a broader, social dimension to individual motivation”. We explore the role of economic resources in the joyful emotional and relational experiences of motherhood. Evaluations of cash transfer programs and related policies must skillfully capture the potential impacts of resources on these multiple and complex facets of the emotional and relational experience of parenting to fully assess such interventions’ consequences for families.

## 1 | Background

### 1.1 | The Emotional Experience of Parenting

When studying parenting, scholars tends to focus far more on negative emotions, such as stressors, than positive ones (for discussion, see Nomaguchi and Milkie 2020). Researchers have detailed the risks of depression, anxiety, and burnout among mothers, finding that parents with lower incomes are in circumstances that heighten their risk of experiencing negative emotions, and that these have adverse repercussions for their children (Mikolajczak et al. 2018; Norcross et al. 2020). Parents’ exposure to stressors is structured, in part, by systemic factors, including racism and sexism. Structural oppression significantly raises the risk of stress exposure for Black mothers and other parents of color (Bonilla-Silva 2019; Paradies et al. 2015). Such risks are heightened for women due to the heavily gendered cultural norms of what “good parenting” is supposed to look like for women (Hays 1996; Lareau 2011) and by their children’s developmental stages (e.g., what it is like to parent an infant versus a teen; Luthar and Ciciolla 2016).

A smaller body of research focuses on positive emotions in parenting. In time-use data, parents report more positive emotions when they are with their children than apart from them (Meier et al. 2018; Musick et al. 2016), with no differences by maternal education (Kalil et al. 2025). Using ecological momentary assessment techniques, Kerr et al. (2021) find that, when they are actively caring for children, parents report more intense positive emotions and a diverse array of positive (e.g., content and joyful) and negative emotions (e.g., frustrated and worried; see also Negraia and Augustine 2020).

Although joy has received attention from psychologists and theologists, among others, they have not landed on a singular and simple definition. Across sources, we see joy involving feeling wrapped up in a positive moment, having a feeling of meaning or purpose in life, and feeling connected to others or something larger than oneself (for discussion, see Johnson 2020; Watkins et al. 2018). People derive joy from the meaning and value they place on their social roles (Krumrei-Mancuso 2020) and in the connections they feel with others (Watkins 2020). We can hear this relational theme of connection come through regarding Black joy in particular, as Sobande and Amponsah (2025) write, “It is the comfort of kinships, the beauty of enduring love, the sharing of ancestral knowledge, and the heartening feeling of home(s)” (p. 403). Some have emphasized the power of Black joy for resilience or resistance (Neville 2024; Waheed 2018). However, others have noted these elements need not be present—Black joy can be apolitical, about healing, or embedded in “moments of mundanity and everydayness in the lives of Black people” (Brock 2020; Sobande and Amponsah 2025, 408).

On the basis of these definitions, joy is central to human flourishing, and particularly so in the context of stressful and oppressive situations. Generally, joy stands apart from some other positive emotions, like happiness, in being more inherently relational and connected to a larger sense of purpose (Emmons 2020). A focus on joy, therefore, aligns with understanding individuals as embedded in networks of relationships.

In the present study, we highlight the joyful emotions that women describe from interacting with their children and in their roles as mothers. As individuals are embedded in family systems, positive emotional impacts on one member ripple through others in the family system and vice versa (Bowen 1993). Previous work finds that positive family relationships can promote resilience for mothers experiencing provider role strain (Mendenhall et al. 2013). These family dynamics provide us with a more comprehensive understanding of the relational nature of intervention impacts. Further, because Stifter et al. (2020) propose that positive emotions emerge early in life and develop in the context of the relationship between child and caregiver, resource distribution interventions that support positive and relational experiences for parents and children could manifest in long-term emotional benefits for them, potential policy outcomes of interest.

Social scientists have recently called for scholarly attention to positive experiences, such as joy, as part of an overhaul of dominant epistemological approaches that treat negative outcomes as the primary focus of legitimate study (Bonilla-Silva 2019; Pattillo 2021; Shuster and Westbrook 2022). In responding to that call, we center positive emotions to provide insight into the complexity of resources that individuals use to improve their families’ ability to not just survive but to flourish. This approach also helps to ascertain the benefits and costs of policy interventions more holistically.

### 1.2 | Subjective Well-Being and Policy Analysis

Largely outside of the pages of the *Journal of Policy Analysis and Management* so far, some scholars have advocated for and explored the inclusion of measures of subjective well-being in policy analysis; this work has especially focused on measures of life satisfaction and happiness (see, e.g., Gedikli et al. 2023; Kim and Kim 2012; Maccagnan et al. 2019; Meynhardt et al. 2024; Oishi and Diener 2014; Pacek et al. 2019; Takahashi et al. 2021; Weijers and Jarden 2013). This scholarship aims to expand the types of outcomes on which policy evaluation is based beyond the more typical economic and human capital measures. Although these perspectives have pushed the policy field forward, they have largely taken an individual approach (as opposed to a relational perspective), been based on researcher-driven indicators of the subjective well-being measures, and focused on hedonic well-being (e.g., life satisfaction, happiness), rather than eudemonic well-being (e.g., joy; for discussion of joy as an expression of eudemonic well-being, see King and Defoy 2020; Neville 2024).^1^ In contrast to hedonic well-being, eudemonic emotions—such as joy—are marked by purpose, meaning, connection in life, engagement, and autonomy. Therefore, to only focus on hedonic well-being is to “neglect important aspects of positive psychological functioning” (Ryff 1989, 1070). Some research shows that eudemonic well-being, but not hedonic well-being, is linked to longevity (Steptoe et al. 2015). Therefore, to draw a more comprehensive picture of intervention impacts, we need to take a relational approach, understand the emotional experiences that policy targets themselves raise as highly meaningful, and attend to aspects of eudemonic well-being. In the present study, we explore mothers’ joy in their parenting—as an expression of eudemonic well-being—and how they may use unconditional cash transfers as a means to create joy.

### 1.3 | Parental Allocations of Economic Resources

A large body of research examines the negative consequences of parenting in a context of cumulative disadvantage (Cooper and Stewart 2021; Duncan et al. 2017). Financial resources are expected to benefit children directly, as well as operating indirectly to benefit children by affecting their parents’ emotions—alleviating parents’ stressors, thereby resulting in more beneficial parenting (Duncan et al. 2017; Shaefer et al. 2018). Research in Zambia and Kenya indicates cash transfers increased happiness among mothers of young children (Natali et al. 2018) and recipients’ psychological well-being but without a decline in stress (Haushofer and Shapiro 2016). Existing studies of unconditional cash transfers in the United States have tended to ask whether these transfers alleviate negative and individual outcomes. The larger randomized controlled study from which the present study draws its data, Baby’s First Years (BFY), has shown that, among mothers in poverty, unconditional cash transfers did not alleviate their stress nor increase their feelings of happiness or agency (Gennetian et al. 2024; Magnuson et al. 2024). However, beyond this one-item measure of happiness^2^ and the scale rating of mothers’ sense of agency,^3^ BFY is limited in what it can tell us about mothers’ relational and positive emotions (especially eudemonic well-being). The present study explores how BFY mothers themselves discuss their positive emotions in the context of their parenting, as we see qualities in these narratives that may not be well captured by a general question about happiness, for example. As an unconditional cash transfer experiment, BFY gives us an opportunity to examine the role of cash resources generally and a cash transfer intervention specifically in these emotional experiences.

Counter to a view of money as an asocial or utilitarian medium of exchange, Zelizer (1994, 2012) emphasizes that money is inherently social and relational, used to build and maintain relationships, and given unique meanings depending on its context. Parents use money to shape their relationships with their children and construct their sense of self, what Zelizer calls “relational work” (see also Bandelj and Gibson 2019). Opportunities for experiencing parenting joys, therefore, meaningfully occur through financial exchanges and events. For example, we can view a mother’s purchase of a coffee shop cake-pop for a child as a food allocation, or we can see it as relational work—a financial allocation that can shape and build family relationships. When we view this as food consumption, we miss the feelings of family bonding and the creation of enduring family memories in such an outing. To integrate this perspective when evaluating income-based interventions helps to ground policy research in a relational perspective (Bogenschneider et al. 2012).

Parents’ desires to provide for children in line with dominant cultural standards often shape spending and the earmarking of additional resources for children (Mendenhall et al. 2012; see also Hays 1996). In addition to spending on basic needs, parents affirm their relationships with their children by spending on items or experiences that bring joy (Mistry et al. 2008; Sykes et al. 2015). Especially for parents with constrained resources, “symbolic indulgence”—buying small, occasional treats that children request—can be important (Pugh 2009). In the present study, we examine how consumption practices are tied up in the joys of parenting that mothers articulate, in connection with their monthly cash gift.

## 2 | Data and Methods

We take a symbolic interactionist approach in this study of the emotional and relational experiences of motherhood (Blumer 1986; Carter and Fuller 2016); this approach attends to how people assign meaning to the subjects, objects, and interactions in their lives. We examine how mothers make meaning of, and therefore experience, their relationships and interactions with their children, including their purchases for and with their children. Our analytic strategy combines aspects of flexible coding for large samples in qualitative data (Deterding and Waters 2021) with the dimensionalization step of dimensional analysis (to understand the variation in how the core concepts presented themselves), which is rooted in the symbolic interactionist perspective (Kools et al. 1996).

We use data from Baby’s First Years: Mothers’ Voices (BFY: MV). BFY: MV is the qualitative companion to BFY, a randomized controlled trial examining how monthly, unconditional cash gifts impact child development (Gennetian et al. 2023; Noble et al. 2021). Recruited in New Orleans, Omaha, the Twin Cities, and New York City, 1000 mothers living at or below the federal poverty line consented to participate in BFY after giving birth and prior to being randomly assigned to receive a monthly cash gift of either $333 or $20. Distributed via debit card, mothers receive the cash gifts for their child’s first 76 months (6.3 years) of life.

### 2.1 | BFY: MV Sample

The data for the present study come from interviews with 80 randomly selected mothers from BFY in New Orleans and the Twin Cities to whom we extended invitations to participate in semi-structured interviews. We stratified our sample to reflect BFY’s proportion of mothers from those metro areas, to equally balance the representation of both BFY: MV gift groups in each site, and to ensure adequate inclusion of first-time mothers. For logistical and financial reasons and to ensure we included an adequate number of participants from both gift groups in each site, we focused this phase of the qualitative study on two of the four sites, which offered a contrast in terms of their population demographics and policy environment; we conducted a separate wave of data collection in the other two sites. We invited mothers to participate in ongoing, semi-structured interviews in either English or Spanish. To protect the identities of mothers, we use pseudonyms here. The current study draws on data collected in the first wave of interviews when the focal children were around 1 year old. Although child age provides context related to parenting, this study is about mothers’ emotional experiences of the interchanges with their children as opposed to observing the reactions of children to their parents, so mothers’ experiences with all their children, regardless of age, are relevant (Table 1).

The mothers in BFY: MV are predominantly women of color, with Black mothers making up 65% of the sample. Fifteen percent of mothers have multiple racial identities, 11% are White, and 9% identify with another race, including Asian and American Indian. Eight percent of mothers are Hispanic. At the time of the first interview, mothers ranged in age from 19 to 42, with a median age of 27, whereas the focal children were between 10 and 21 months, with a median age of 13 months. At Wave 1, a little over half of mothers reported having a romantic partner, the majority of whom were co-residential. About two out of every five mothers were formally employed. Most mothers received SNAP benefits (74%), WIC (64%) benefits, or both; very few received cash welfare benefits (TANF) (9%) or formal child support (9%).

We center the experiences of Black and brown mothers in this article, as they compose the majority of our sample. The emotional experiences we study are systemically shaped by intersecting forms of oppression—not just individually derived (Collins 1987, 1994; Dow 2016). All women in the study are parenting in a racialized system, which weighs far more heavily on women of color (Bonilla-Silva 2023; Williams and Baker 2021) but can also adversely affect White women whose socioeconomic circumstances place them in a marginalized social position (McGhee 2022).

### 2.2 | Data Collection

Four locally based interviewers spoke with mothers at Wave 1. Three identified as women, three as people of color and one as White, and one as a mother. All had a college degree or more. The first wave of interviews began in July 2019, occurring in person (68%) until March 2020 when we transitioned to phone interviews (32%) in response to the COVID-19 pandemic. We completed Wave 1 interviews in September 2020. The onset of the pandemic significantly shifted the context in which mothers parented, from the demands of social distancing that increased the time spent at home to the disruption of early care and education, school, employment, and income. Because most BFY: MV mothers were not formally employed prior to the pandemic’s onset, employment loss was less of a risk for them than for other women their age who had not recently had a child. Research suggests the pandemic placed immense stressors on parents but also presented opportunities for joyful time together as a family (Milkie 2020). The Wave 1 interviews were also in progress at the time of George Floyd’s murder on May 25, 2020, in Minneapolis, one of the BFY: MV sites, and the widespread protests against police violence that ensued; police brutality has long affected motherhood for Black mothers, including mothers in BFY: MV, though it gained heightened salience at that time, especially for the mothers in that community.

Lasting on average 1.5 h, the semi-structured interviews spanned topics of family life, parenting, work, and money. Relevant to the present study, we explicitly asked mothers about what they experienced as the stressful parts of motherhood and about some of their favorite aspects of motherhood: “What are some of your favorite parts of being a mother?” “What are some of your least favorite or most stressful parts of being a mother?” In addition, because these were semi-structured interviews, emotional experiences related to parenting could arise throughout the interview, not just in response to these specific questions.

We attended to the role of interviewer race as the interviews occurred. Given cultural norms that often push a “strong” image of Black mothers (Dow 2015; Elliott and Reid 2016), we were attentive to whether mothers would feel comfortable being vulnerable in the interviews, revealing weaknesses or challenges, even to someone from a different racial or ethnic group. We did often see vulnerable revelations. We believe that the mothers’ vulnerability is a testament to the work the interviewers did to build rapport, which could require greater effort to build trust between people of different races. The mothers’ vulnerability may also reflect their desire to have their voices and lived experiences be heard.

### 2.3 | Data Analysis

Our research team includes four co-authors with research experience across varied methodological approaches; we all identify as women and have graduate degrees, and two of us are mothers. The first author identifies as White and has engaged in an array of qualitative interview research projects, most commonly with parents who have limited incomes. The second author, who identifies as White, is a doctoral student with prior experience in early childhood policy and practice and conducted interviews for this study. The third author is an African American faculty member who has engaged in extensive community-based participatory action research with mothers living in predominantly African American communities. The fourth author has done research on the social meaning of money for parents. Our team drew on our personal and professional experiences in reflecting on the interviews throughout the data collection, coding, and analysis process.

Our analysis involved three main steps. First, we used the qualitative data software, *Dedoose 9.0*, to index the transcripts—primarily made up of deductive coding in which codes are derived from the content of the interview guide and existing literature. In their flexible coding approach, Deterding and Waters (2021) prescribe this initial step when working with large qualitative datasets as a means to organize and become familiar with the data. For the remainder of the analysis, we focused on excerpts from two index codes—*Stressful parts of motherhood* and *Best parts of motherhood—*to explore mothers’ emotional experiences of parenting.

Next, to analyze mothers’ emotional experiences, we undertook a process of dimensionalizing that “entails the designation or naming of data bits and the expansion of those data into their various attributes” (Kools et al. 1996, 316). For every mother in our sample, we analyzed all identified excerpts and then developed a set of subcodes delineating both negative and positive emotional experiences mothers portrayed. Three members of the research team reviewed excerpts together for consistency in the conceptualization and application of these subcodes. Examples include managing daily family life, receiving love and affection, and children growing and succeeding. As mothers related specific instances and stories, we analyzed what these experiences and interactions with their children symbolized to mothers. We note that just because a mother did not mention a particular stress or joy did not mean it was not an emotion she experienced, but rather it may not have been top of mind for her in recounting her experiences as a mother. Furthermore, there is an array of contextual factors that may play a role in mothers’ experiences of stressors and joys, such as their employment situations or relationships with their child’s other parent, which we incorporate in our discussion below.

To identify dimensions within mothers’ narratives, we took an abductive approach to coding, refining our set of subcodes—consolidating or redefining as appropriate—and examining relationships between them, working iteratively as a team to arrive at consensus. It was during this stage of analysis that we labeled these positive experiences mothers described as examples of joy because they commonly included its core emotional and relational elements—such as feelings of connection and purpose in life through mothering, elation, and being together as a family. We then overlaid mothers’ experiences of joy with elements of eudemonic well-being—meaning and purpose, engagement and connection, and autonomy—elucidating how joy serves as an expression of eudemonia. We note that although the present study is focused on mothers’ joys, we examine this emotion without disregarding their stresses.

Our coding approach allowed us to reveal themes we may not have anticipated or known to look for. Our research team worked together to discuss the coding and development of findings to try to ensure that we uncovered potential blind spots or misinterpretations. For example, in discussing the challenges some mothers noted in getting out the door in the morning, we discussed the additional layer of stress that racism and threats of police brutality could overlay on the mundanities of the morning rush to get out the door on time. There were specific discussions about being Black, from an insider perspective, and the role it may have played in certain responses from mothers. Most fundamentally, throughout this article, we strive to center the perspectives of the mothers in BFY: MV, as aspects of their parenting experiences may differ from our own.

In presenting the findings, we first create a baseline understanding of the joys women raised in their narratives about being mothers. We next show how mothers discussed the BFY money in connection with creating joys when raising and mothering children. Throughout, we discuss how the data we present are reflective of the core elements of a joyful sense of eudemonic well-being—purpose, meaning, connection, engagement, and autonomy; we attend to whether particular elements arise in mothers’ financial allocation decisions. These findings make the case for the importance of including positive emotional and relational outcomes tied to eudemonic well-being in studying income-based interventions.

## 3 | Findings

### 3.1 | Joys of Mothering and Eudemonic Well-Being

We enumerate the ways women reported experiencing joy in motherhood in Table 2. We use these to establish the experiences that undergird the emotions mothers described, including through their financial allocations, which we analyze below. Many of the joys mothers described emerged within family-building activities similar to those of Tubbs et al. (2005) detail in their study of mothers with lower incomes. In their study, mothers viewed activities, such as talking together, eating together, playing together, and sharing treats as opportunities to build strong family connections and relationships during regular family life.

Each of the three most common joys of motherhood was described by more than a third of the women: spending time with children (38%), children growing and succeeding (35%), and receiving love and affection (34%). These findings underscore the multifaceted nature of the joys of parenting and how various experiences with their children may hold related but distinct meanings for mothers. The first of these kinds of joys emerges from investing time in the mother–child relationship. The second of these joys comes from witnessing and, perhaps, facilitating children’s development. The third centers mothers’ joy from how their children treat them. As Table 2 illustrates, there are many other experiences in which women also find joy in their mothering.

Jayla, a Black mother of one in New Orleans, speaks to the joys of quality time and celebrating children’s growth. She enjoys describing the changes in her daughter’s personality, recalling, “When she was a baby, she was chill and very playful like she still is now And now she’s even more active, and she talks a lot for most 18-month-olds She surprises us every day with a new word.” Jayla says her favorite parts of being a mother are: “Watching her learn new things. And, just playing and interacting with her.” Aligned with the definitions of Black joy we discuss above, Jayla articulates how feelings of connection with her daughter, getting to spend quality time with her daughter, and feeling a daily sense of wonder at her daughter’s accomplishments are joyous parts of motherhood for her. These feelings also clearly speak to elements of eudemonia, displaying the sense of connection, engagement, meaning, and purpose Jayla derives from mothering.

Houa, an Asian American mother of six in the Twin Cities, reports relishing every stage of motherhood: being pregnant, breastfeeding, and now, “just watching them growing up, that’s what I enjoy.… They were just babies with little tiny feet.… Time passes, and it’s just like, wow, they’re so big now.” At this point in building her relationship with her youngest, Houa describes the challenges and joys of his developing personality. “He’s very sweet and also very demanding. He has a sense of humor about him.… If he wants something, he’ll say it .… He’ll signal it or just starts crying because he doesn’t even know how to speak in complete sentences yet, but if he starts screaming and pointing at it then I know that he wants something like that.” While laughing, Houa recounts how her youngest is determined to do what he wants. “He’s very persistent of being naughty. … He knows the word ‘no,’ so I’m like, ‘No.’ But he likes it because it’s fun .… ” Parenting a toddler can be difficult, but Houa also experiences joy in seeing his personality come through in their interactions. From Houa, we hear her awe in witnessing all the stages and the fine details of her babies learning and growing from infancy through childhood. Houa’s positive emotions about motherhood arise from the role itself and the mix of her children being “demanding,” “sweet,” and “fun.” We hear about her eudemonic joy—her sense of purpose, meaning, and engagement—from her interactions with her children.

Patrice, a Black mother of five from New Orleans, expresses a profound sense of well-being from receiving love and affection from her children. When we ask Patrice about her favorite parts of being a mother, she tells us, “The love you get, the unconditional love. A little person that love you. Unconditionally too. That’s my favorite part. And when I can make them smile.” Here, Patrice describes how “unconditional love” from her children satisfies a key human need (Baumeister and Leary 2017) and speaks to the sense of existential recognition Neville (2024) articulates as a component of Black joy. Patrice describes experiencing a deep sense of fulfillment from feeling loved by her children and creating moments of joy in their lives; for her, mothering can create eudemonic well-being by inducing feelings of purpose, connection, and engagement.

### 3.2 | Joys Across Groups

Although some of the joys of parenting that mothers described required resources, such as time to spend with their children or money to materially provide for them, the presence of financial struggles did not make for the absence of joys. To ensure that we were not just seeing joys reported by mothers without any substantial stressors, we also coded and tallied the stressors of parenting that mothers described, like we did for joys (see Table A). Using these counts, we saw that, for example, among the 31 mothers who describe provider role strain (getting by on a low-wage job or limited income) as one of the stressful parts of motherhood, eight (26% of these mothers) tell us about the joy they find in spending time with their kids, eight (26%) explain how their children help regulate their emotions, 10 (32%) describe the love and affection they experience with their children, and 13 (42%) talk about the uplift they feel from seeing their children grow and succeed. This illustrates the ways in which joys and stressors can coexist and are not mutually exclusive, suggesting that an intervention could conceivably affect one but not the other (e.g., could increase joys without easing stresses).

We see mothers from both the large- and small-cash-gift groups richly discussing the joys of motherhood.^4^ Because we do not assess the frequency or intensity of mothers’ joys, we cannot say whether those in the large gift group experience joy more often or more strongly than their counterparts in the smallgift group. What we focus on in our analyses below, therefore, is how mothers’ narratives of their joys help us understand their financial allocation decisions within the context of a cash transfer intervention. In doing so, we see how, even though mothers often described parenting joys regardless of gift group status, it was mothers with the larger BFY cash gift who more commonly described spending those particular dollars in pursuit of parenting joys.

### 3.3 | Joys and the BFY Cash Gift

We see that mothers deploy the BFY money—especially the larger gift amount—to shape the joys of motherhood, though there is considerable variation among BFY: MV mothers. Most notably, we see how the eudemonic element of autonomy arises more commonly in mothers’ discussions of joys in their financial allocations, compared to the joyful family experiences mothers described above. In their narratives, we could hear the joy that some mothers experienced when using the BFY money to provide the basics as well as extras for their children. Nina, a 26-year-old, Black mother of four from New Orleans, describes spending the first $333 she received right after her youngest was born. “We got food, a lot of food. … I even went and got the kids a gift. That’s how happy I was. … So, I even got them a toy at the store. I got some cleaning supplies to make sure it was really sanitary for when I brought [my newborn] home.” Nina’s attention to providing the basics—food, cleaning supplies—doesn’t push aside her glee at being able to do something special for her older children. The first BFY payment came at a time of crisis for Nina and so felt transformative because home is a place of rest and resistance in Black culture and Black joy (hooks 2009). Purchasing items for their children allowed mothers like Nina to take care of their children in the way they desired, bringing them joy. The investments in joys mothers described making with BFY were often relational, and we hear how Nina feels like she is fulfilling her purpose as a mother as she deploys the money to care for her children in multiple ways. In Nina’s description, we hear the eudemonic elements of purpose and autonomy as she recounts her joy making these spending decisions.

Patrice, who we met above, gestures around the room during the interview, pointing out how she has used the $333 in monthly BFY money for baby Demyah, including a bassinette, swing, and walker. Except, she says, both resigned and loving, “She don’t like none of it. … She likes for me to hold her.” The money offered Patrice an opportunity to equip her infant daughter with a sleeping space and other tools, even if it is her mother’s arms that Demyah most wants. We see how resources play a role in Patrice’s emotional experience of motherhood, with her allocating BFY dollars to provide her daughter with what she sees as the furnishings of a good childhood; her spending, therefore, is an act of mothering. In her narrative about her allocation decisions, we hear about the eudemonic elements of connection, engagement, and autonomy.

Some mothers use the BFY money to shape experiences for or with their children. Nina finds joy in her children’s enthusiasm for the world and uses the BFY money to create experiences that draw out their excitement. “It’s small things that excite them.… And they’re even like, you know, ‘Mama, can we get popcorns so we can have movie night?’” This little treat is exciting for her kids. Faith, a 31-year-old Black mother of five, occasionally treats her children to dinner with the BFY money. We hear about her eudemonic sense of purpose, connection, engagement, and autonomy in these joyful outings, as she says, “I check my email and it says loaded. ‘Oh, okay. I have some money. Y’all, let’s go eat.’ They’re like, ‘For real Mom, we’re going out to eat?’ ‘Yeah, we’re going out to eat.’” She tells her children to choose foods that’ll be interesting, beyond their beloved macaroni and cheese, and different from what she can cook at home. We see here the relationship building that Nina and Faith are doing with the BFY money, aligned with the focus on kinship in the literature on Black joy. From the perspective of scarcity alleviation, we might see spending on food as possibly reducing food insecurity. However, these mothers’ narratives reveal how they are using the money on food to build stronger bonds and memories with their children, with Nina’s family curled up together for movie night and Faith’s kids sharing the experience of broadening their culinary horizons during a family outing.

Victoria, a 30-year-old mother of four from the Twin Cities who identifies as Black and American Indian, was in disbelief when she found out she would receive $333 each month when her son, Micah, was born. After buying special items for Micah when he was a newborn and then using it for some household bills, Victoria developed a monthly family ritual in using the BFY money with her children, taking them all to the store; this seems to create joy and reinforce the eudemonic elements of engagement and autonomy for her. “I’m like, ‘Well, let’s go get [Micah] some stuff.’ … [T]hen I was like, ‘Well, how about everybody gets something too?’” Victoria engaged her older children and treated them to something special while also spending the cash gift primarily on Micah. She says when the BFY money comes on the designated day, “[The children], like, all are ready. Like, we go to the store and they, like, get stuff.” In her narrative, we hear the emphasis on how—over and above the items purchased—the BFY money created opportunities for her to build relationships in her family in ways that she views as valuable—developing a meaningful ritual around the family spending time together each month.

For some mothers, the BFY money plays an important role in their budgets that can directly and indirectly facilitate the joys that mothers describe experiencing. When Houa, who we met above, found out she was pregnant with her youngest, she was concerned because “I was not financially stable.” When she recounts her first experience with BFY, she shares, “First it was too good to be true, but when the money actually showed up, ‘Oh, my gosh. It showed. They’re actually doing it for real.’ I was so happy. … I get to buy clothes for [the baby]. The money helped out a lot because at the time I didn’t have a job.” Now, a year later, Houa is working full-time. She describes how she currently allocates her income and uses the monthly $333 BFY gift: “I usually save that money because I already have enough. And I have another paycheck that covers the bills. So, that money goes into a savings for the baby to use if he needs a new car seat, if he needs a stroller, if he needs diapers, wipes, clothes.” In comparing how Houa first experienced and used the BFY money to how she does now, we can see how it was enormously useful to her when she was experiencing the stress of a financial crisis. Now, Houa feels good about being able to comfortably meet her baby’s needs with the BFY money. We see how, even with Houa’s evolving financial situation, she continues to enact her view of good motherhood by earmarking BFY money for savings for her baby’s future needs and how such spending facilitates her joyful eudemonic feelings of autonomy and purpose.

Deja is a 23-year-old Black mother of one in New Orleans. Among the many joys she experiences in motherhood, she delights in having time with her son, Lucas, and in the accomplishment of being a good parent. To her, part of being a good parent is providing materially for her son, and in her descriptions, we hear about the sense of purpose, meaning, and autonomy that she derives from mothering. She shares that “I just feel like I’m letting my baby down if I wasn’t able to buy him something that he really wants.” To feel accomplished as a parent, it’s important to Deja to be able to meet both her son’s needs and wants. Deja says her current retail job would not allow her to do that, so it is the BFY money that makes it possible. “So, it really helped me to not to get a second job. To stay at home more with my child.” For Deja, the BFY money means that she does not face a trade-off between physically being with and providing for her son in the way she wants. Rather, Deja has more time with Lucas and can feel accomplished in her parenting because she can provide for him without spending long hours away at a second job, which brings her joy. Her descriptions of her feelings of bonding with her child and self-determination align with the conceptions of Black joy we review above.

In the BFY randomized controlled trial, mothers in the large- gift group spent significantly more on child-focused expenditures (Gennetian et al. 2024). Although stories of how the BFY money facilitated mothering joys were common and impactful among the mothers in the large-gift group, mothers in the small-gift group also sought these opportunities. For example, Jayla, who we met above, uses the BFY money to buy things her daughter needs (like toys and learning games), for toddler snacks, or for “little knickknacks,” treats Jayla knows her daughter will enjoy. Although she only gets $20 a month from BFY, she still uses this money in a relational way, to add enjoyable items and experiences to her daughter’s life. Such purposes provide Jayla with feelings of meaning and autonomy.

From the experiences of women in BFY: MV, we observe several ways in which money enables joy in motherhood. As the stories above illustrate, money can be used to access opportunities for delight, fulfillment, and bonding, though the specific allocation decisions mothers made to evoke these emotions varied across families. It is important to note that the unique meaning mothers ascribed to the BFY dollars—“the baby’s money”—likely shaped the kinds of allocations and, therefore, joys they experienced. That is, BFY did not totally meet families’ financial needs and therefore enable the pursuit of these special kinds of spending; rather, because mothers mentally earmarked this money as being for their children (Halpern-Meekin et al. 2024; Sykes et al. 2015; Zelizer 2012), they spent a portion of it in child-focused ways that seemed to bring parenting joys.

In this analysis, we see that stresses and joys are not just two sides of the same coin. Camille, a 26-year-old Black mother of two from New Orleans, used the BFY money to get her electricity turned back on. She expressed relief, not joy, when recounting this event, in contrast with the experiences of mothers like Nina at family movie night or Deja letting her baby pick out a toy at the store—they share their elation, not their eased hardship. We can observe the ways in which money affects stresses and joys distinctly; we cannot infer the types of joys mothers experience from the stressors they say are relieved by the BFY money. Furthermore, situations that bring about joy for mothers may still give rise to stress. Family dinners out like those that Faith told us about and shopping excursions like those Victoria and her children take part in are meaningful sources of joy but are not necessarily stress-free. Spending on these types of activities presented the possibility of common stresses mothers reported as part of parenting for them—getting everyone ready and out the door or managing sibling arguments about where to go or what to get. Although stresses can arise when using cash transfers in these ways, mothers do not sacrifice the joy their families derived in order to avoid stresses. Thus, we must attend to positive and negative emotions distinctly in evaluating policy impact to understand how they might be shaped by material resources and related interventions.

The relationship between parent and child is reciprocal for many, often centered on the time they spend together and the joys this time together can bring. Parenting can be stressful, and not having enough resources is a source of stress, but this does not mean that the relationships parents have with their children are a source of stress. Instead, we hear about the deep and meaningful connections that mothers experience such as feelings of unconditional love and caring intensely for someone—both emotionally and physically—and fostering their growth; that is, we hear multifaceted presentations of the joyful elements of eudemonic well-being coming through in mothers’ narratives. The relational spending mothers do with their BFY dollars flows from this context; to fully understand such an intervention, therefore, requires attention to relational impacts.

## 4 | Discussion

In this study, we show what kinds of joys women find in mothering and how they deploy financial resources in relational ways to invest in experiences that offer joy. We present a multifaceted understanding of mothering for women with limited resources and from marginalized groups. This makes the case that to fully understand the impact of interventions, such as cash transfers, our assessments must include whether they elevate good outcomes (e.g., joy), not just diminish negative ones (e.g., stress); the latter has often been the focus of research on parents with lower incomes (McLoyd 1990; Norcross et al. 2020; Sharlin and Shamai 2000). This approach is in line with recent work that centers joy in research with people from marginalized groups (Baker 2021; Lu and Steele 2019; Shuster and Westbrook 2022; Stewart 2021) and advocates for a relational perspective on policymaking (Bogenschneider et al. 2012). Particularly when the pressures of mothering (Hays 1996) come layered on top of managing structural oppression, the importance of finding joy should not be undervalued (Bonilla-Silva 2019; Collins 1987). Building on research advocating for the inclusion of subjective well-being in policy analysis (Dolan and Metcalfe 2012; Hicks et al. 2013; Stiglitz, Sen, and Fitoussi 2009), we additionally show the value of attending to eudemonic and relational experiences, such as joy, and learning about the shape and texture of these emotional experiences from policy targets themselves. As cash transfer programs grow, these lessons should be incorporated into evaluations of them. With this in mind, we draw out three implications of our findings.

First, we find that the aspects of motherhood in which women take joy are varied and multi-faceted. This suggests that the types of simple measures often used to assess the presence of positive outcomes in poverty interventions (e.g., single-item life satisfaction or happiness scales; for summary, see McGuire et al. 2022) may be inadequate to capture the nuanced nature of people’s positive experiences in these programs. Even measures that use a wider range of items are often individualistic, not relational, in the aspects of quality of life they assess (e.g., health, personal future prospects, locus of control; Kilburn et al. 2018). The RCT arm of the Baby’s First Years study includes a set of positive measures—general feelings of happiness, agency, and parenting competence (Gennetian et al. 2024; Magnuson et al. 2022); these are distinct from the relational joys that emerge in the qualitative narratives. Although the RCT showed no impacts on these outcomes of happiness, agency, and competence, the joys mothers experienced from some of the ways they deployed their cash gifts with and for their children came through strongly in their qualitative narratives. In recent work from the Rx Kids unconditional cash transfer to pregnant and postpartum people in Flint, Michigan, researchers included five items assessing mothers’ feeling loved, having hope, feeling respected, feeling valued, and feeling empowered (Hanna et al. 2025). Those receiving the cash transfers reported statistically significantly higher levels of these emotions in a difference-in-difference analysis. This is suggestive of the insights that could come from including an array of positive emotional outcomes in evaluation research.

Beyond cash transfer studies, when we look at the data infrastructure poverty scholars often depend on to conduct their research, we see a heavy slant toward measuring negative outcomes in data sets like the Panel Study of Income Dynamics and the National Longitudinal Survey of Youth. As the present study shows, relationships with children are an immense source of joy for mothers; not tapping into this relational context means risking an incomplete picture of their emotional experiences and, therefore, policy impacts. In both policy-specific studies and in building future data infrastructure, therefore, more robust attention to positive and relational outcomes would be useful. This shift in approaches suggests the benefits that could come to the policy analysis field of starting to ask questions about a more extensive set of potential outcomes. Considering an expanded set of outcomes could lead to the use of methods from other fields that allow us to ask and answer new policy questions. (For example, physiological measures of serotonin or oxytocin to assess positive emotions; ecological momentary assessment [Shiffman et al. 2008] to track well-being and emotional experiences over time and by situation—for example, do emotions and relationships fluctuate depending on the timing of SNAP receipt, which varies in its distribution schedule across states?) As a starting place, psychology researchers have developed measures of joy that policy researchers could explore (e.g., Watkins et al. 2018).

Second, we learn how mothers deploy financial resources in ways that align with the aspects of parenting that bring joy for them and their children. Therefore, even if cash transfers are not adequate to alleviate stresses (Haushofer and Shapiro 2016; Magnuson et al. 2024), the present study raises the possibility that they could still facilitate families creating joy. We hear from Patrice about furnishing her daughter’s infancy with a baby bed and swing and from Houa about buying clothes for her newborn. In these examples, we show the relational opportunities that having the BFY money created, in which women can enact motherhood, or experience success in the provider role, in line with their parenting values and self-definition (Bandelj and Gibson 2019; Mendenhall et al. 2013; Zelizer 2005). This can enable features of eudemonic joy, such as connection, purpose, meaning, engagement, and autonomy. Other forms of resources, like in-kind benefits, may not similarly spur or even allow such child-focused spending and, therefore, the parenting joys mothers told us about from their monthly cash gifts. In particular, the feature of feelings of joy from autonomy may be more likely with unconditional cash transfers than in-kind benefits, as it allows the policy targets themselves to make allocation decisions (Halpern-Meekin et al. 2024).

Third, many of the joys of mothering that women describe are embedded in their relationships with their children—loving each other, witnessing and facilitating children’s development, and bonding through shared experiences. This aligns with eudemonic understandings of joy in general and with Black joy in particular, which emphasize the connective, relational, and purpose-filled nature of these experiences. We see mothers’ experiences of joy as important in their own right; additionally, their joy may influence their parenting, as well as their children’s development. Following the broaden-and-build theory of positive emotions (Conway et al. 2013; Fredrickson 2001), the experience of joy in motherhood may shape mothers’ cognitive processes, widening the scope of their attention, encouraging flexibility in their thinking, and promoting creativity in their problemsolving. Mothers could mobilize this shift in cognitive processes to serve them in their parenting. Further, mothers’ sharing joy with their young children plants the seeds for such positive emotions in their children as they grow up (Stifter et al. 2020). By financially investing as they do in shared positive experiences, such as family movie night or a special family meal, mothers are creating “co-experienced positive affect,” which Brown and Frederickson (2021, 58) maintain create “the origins of social skills, social bonds, and caring, healthy communities” by building relationships and contributing to socioemotional development. There may be long-term payoffs as parents build these relational bonds and resources for themselves and their children, even as they navigate intersecting forms of oppression (Collins 1987, 1994; Dow 2016). Further, scholars have found that outings and time spent together contribute to sustained family bonds and positive family memories, which are associated with families’ quality of life, including family cohesion and adaptability (for discussion, see Agate et al. 2009; Hornberger et al. 2010; Shaw et al. 2008; Wang 2008; Zabriskie and McCormick 2001). Future policy analysis research could draw on measures from other fields to quantitatively assess impacts in these areas (e.g., family life quality, Rettig and Leichtentritt 1999; family environment scale, Sanford et al. 1999; family adaptability and cohesion evaluation scale, Olson et al. 1983).

As with all research, our study has its limitations. The aim of our qualitative study is to broaden our understanding of a cash transfer intervention as an example of incorporating attention to positive, relational outcomes in policy analysis. The nature of our sample means we cannot say whether the joys parents of other genders, socioeconomic positions, or regions of the US experience would be similar to those we see here. Our sample size and data collection method also limit our ability to deeply explore and detect many patterns in joys between subgroups. Further, we only focus on those joys of motherhood that women raised, as opposed to providing a complete accounting of all possible sources of joy women might endorse from a comprehensive list if responding to a survey. Like child–parent relationships, joys may change across the life course of the mother and the child; what we observe here is not what a mother might report at a different point in time, such as when her children are teenagers (Luthar and Ciciolla 2016). There are also other purposes to mothers’ spending, beyond cultivating joy as we examine here, such as developing children’s human capital (Lareau 2011) or socialization into a racialized world (Dow 2019). More broadly, we have not explored perspectives that criticize consumer culture or the commercialization of childhood (for discussion, see Pugh 2009) or that examine racial differences in access to consumption opportunities (Charron-Chénier et al. 2017), as they are outside the bounds of the present study, though nonetheless important. Finally, we are using one policy lever—cash transfers—to make a broader case for shifting our orientation to studying policy impacts; in select other areas of policy, it may make less sense to attend to positive emotional or relational outcomes (e.g., banking regulations).

The present study advances our understanding of the moments mothers experience as joyful, how those moments appear in their day-to-day lives, and the abundance of joy we see in the lives of the sorts of mothers whose deficits often receive the bulk of research attention. The study also highlights the ways that Black joy is present in mothers’ self-determination in their spending and parenting decisions (Neville 2024) and in the dailiness of their efforts to build the family life they desire for their children (Brock 2020; Sobande and Amponsah 2025). This is a particularly important undertaking with the women in our study because Krumrei-Mancuso (2020, 59) cautions that “theories and measures of joy may lack sensitivity among populations and individuals who have less privilege, opportunities, education, freedom, or resources.” Future research among those who study joy could attend to the role social structures, and policy in particular, play in shaping individuals’ emotional experiences; the present study suggests the importance of a relational perspective, not an individualistic approach, to understanding joy.

Research in policy areas such as WIC, government-funded relationship education, after-school programs, and child care programs have found that attending to the ways interventions shape relationships among participants, between participants and staff, and with participants and others in their lives reveal aspects of program implementation, function, and impact that scholarship focused on individual outcomes misses (Barnes et al. 2023; Barnes and Nolan 2019; Halpern-Meekin 2019; Randles 2014; Small 2006). Programmatic, individual, and familial resources can be invested in building and maintaining relationships. In the present study, we see how this includes money enabling parents and children to spend time together and parents to purchase items and access settings for family activities. A thorough accounting of policy implications must include these relational investments and their attendant emotions (including both hedonic and eudemonic feelings).

For cash transfer studies, if the approach taken is only to consider whether the unconditional cash transfer eases emotional and financial stresses and not whether it helps mothers lay foundations of joy—like a child feeling seen and special with the purchase of a dollar store toy (see Pugh 2009)—then the study may be at risk of underestimating the effect the money could have on parents and children (Natali et al. 2018). Joy is not the absence of stress, and so we cannot assess the impact of an intervention on joy by just ascertaining whether parents report an alleviation of stressors. Thus, moving forward, cash transfer studies could measure and explore whether mothers receiving higher gift amounts are more likely to report joyful parenting experiences (including variation in their intensity and frequency) or feelings of family cohesion.

As cash transfer studies inform policy debates (e.g., a monthly Child Tax Credit in the United States), it is essential to have nuanced and well-developed ways of assessing their impacts on parents’ emotions and family lives. As the broaden-and-build theory suggests, there could be long-term payoffs, both for individuals and their children, to bolstering positive emotions in the present (Conway et al. 2013; Fredrickson 2001; Stifter et al. 2020). Further, investing in opportunities for family bonding and building family memories may strengthen families’ quality of life, including cohesion and adaptability, over the long term (Hornberger et al. 2010; Zabriskie and McCormick 2001). Observing the ways in which women derive joy from motherhood helps to capture the multifaceted nature of their parenting experiences. Often, mothers with limited resources or from marginalized groups are discussed from a deficit perspective in comparison to White and more resourced mothers. A fuller treatment of mothers’ experiences recognizes their individual and contextual challenges but does not treat these challenges as erasing their autonomy and potential for experiencing personal and familial joy. We suggest this perspective can contribute to more comprehensive scholarship and informed policymaking. As policies and programs invest in fostering families’ survival, we argue that program success can also be evaluated by whether they foster families’ abilities to flourish.

## Supplementary Material

Appendix 1 Table A: *Stresses of motherhood (N=80)*.

Additional supporting information can be found online in the Supporting Information section.

## Figures and Tables

**TABLE 1 |:** Demographic characteristics of BFY: MV mothers at Wave 1 (*N* = 80).

	N	Percent	Median	Min	Max
**Gift amount**					
High	40	50			
Low	40	50			
**Site**					
New Orleans	50	63			
Twin Cities	30	38			
**Age**					
Mother (in years)	80		27	19	42
Focal child (in months)	80		13	10	21
**Ethnicity**					
Hispanic	8	10			
Non-Hispanic	72	90			
**Race**					
Black	52	65			
Multiple	12	15			
Other	7	9			
White	9	11			
**Motherhood experiences**					
Mother’s number of children	80		2	1	6
Focal child is mother’s first child	23	29			
**Romantic partner status**					
Has romantic partner	45	56			
Co-resides with romantic partner	32	40			
Romantic partner is focal child’s father	37	46			
**Household size**					
Full-time residents only	80		4	2	12
Full-time and part-time residents	80		5	2	12
Lives with member of an older generation	25	31			
**Employment status**					
Formally employed	30	38			
**Program and benefit receipt**					
SNAP	57	71			
WIC	51	64			
TANF	7	9			
Child support	7	9			

*Note:* We provide these tabulations for context. Data regarding formal employment are missing for one mother. Percentages are all calculated using the full sample size of 80 mothers as the denominator. *Source:* Authors’ tabulations.

**TABLE 2 |:** Joys of motherhood (*N* = 79).

	*n*	Percent	Illustrative quote
Spending time with children	30	38	I mean everything about being a mom is fun to me. We have family night. It’s either movie, UNO, or Go Fish. … They’re mine and I have fun with them.
Children growing or succeeding	28	35	The moments that you get to watch them learn and see them just develop as a human being it’s really quite amazing to watch.
Receiving love and affection	27	34	Being there for them when they hurt their self, building their self-esteem, having someone who loved you unconditionally that don’t see no wrong in what you do just love you like automatically, and having the love for another human being, other human being more than you could describe. I love being a mom.
Making children happy or seeing them smile	21	26	Really, waking up in the morning. … The first thing they do is smile at me. … All the, you know, fun we have letting her walk around the house getting into stuff.
Children transforming mother	18	23	[I]t was unexpected when I had my first baby, but I feel like he changed me like as a woman, like you know. Like I had somebody to really be responsible for 24/7. I had somebody who really loved me unconditionally for me.
Children’s unique personalities	18	23	She’s funny. Like I never had a baby like her before. She really, really funny. Like and then I be telling like, an old lady is trapped inside her. Because she do stuff that make you just, you’re open your mouth and say wow.
Providing for and taking care of children	17	21	I like that part like how my kids hold me accountable, you know? And just the responsibility of being a mother. Yeah okay, I have to do this and this and this for my kids because my kids need this and this.
Contributing to children’s growth and development	15	19	I mean, just being able to watch my children grow and being able to teach them things and to show them, you know, how to love and how to express themselves. It’s just everything. I mean, I don’t even know how to sum it up into words. But I love being a mom.
Children regulating mother’s emotions	14	18	I would say the best part is for me, which is just having them. You know, they’re my happiness when things are like crumbling.
Being there for children	13	16	When they’re upset, I like to give them one, pat them on the back, or be the one to tell them it’s going to be OK. Like I love it. And I love the fact that they can come to me, ‘Momma, what about this?’ and ‘What you think about this?’ And I love it. I just like to see my children happy. Because I wasn’t – I didn’t get to have it.
Children’s interactions with one another	9	11	When the kids saw him and held him, that melted my heart, because they just fell in love like I did. I saw it in their faces, you know. I was like, ‘I know, right?’ And it was like, yeah, and they’re such a great help with him.
The accomplishment of being a good parent	7	9	But they’re all happy kids, they’re always happy. … [N]ow, since I’m a single mom doing it all on her own, I love that I’ve done all of this for them by myself.
Relationships with children offer protection from social poverty	6	8	The favorite part is that – well, most of it is I don’t feel alone. That I have my kids and I’m able to – you know, that’s what makes me move on. That I’m there for them. And they’re there for me.
Breastfeeding	4	5	My favorite part of being a mother is breastfeeding, definitely the bonding.
Children’s excitement about and enthusiasm for the world	2	3	I try to let my children enjoy life as much as possible, like even though we don’t have much, even if we go outside, I put a blanket out, oranges and sandwiches, and we, the smallest little things excite them. And that’s what makes me feel like I’m doing a good job.
Other people recognizing what great parents they are	2	3	I educate her the way she talks and the stuff that she knows, [so] a lot of my friends say that they’re going to send kids to me because I teach [my daughter], like I teach her a lot.
Miscellaneous	9	11	It was more exciting with [my younger daughter] just because I think it was just like, we already had a family, just we were growing the family…
No joys	1	1	I don’t really know. I guess like just – I don’t know.

*Note:* One mother was not asked about the joys of motherhood during the interview, which accounts for the difference in sample size between stresses and joys. *Source:* Authors’ tabulations and compilations.

## Data Availability

Because the data this study uses include personally identifying information, they are not currently available publicly.

## Footnotes

1 Eudemonic well-being does receive mention in summative discussions of this field (as opposed to empirical work); see, for example, Dolan and Metcalfe (2012), Hicks et al. (2013), Stiglitz et al. (2009).

2 “Taken all together, how would you say things are these days, would you say that you are very happy, pretty happy or not too happy?”

3 Example item: “In general, I meet the goals that I set for myself.”

4 Looking across location, racial and ethnic groups, first-time mothers versus others, and those we talked to before versus after the onset of the pandemic, we do not see strong patterns across groups in the joys mothers described. This suggests the joys of mothering were not restricted to any one group.
