# Supplementary material for: “When I Can Make Them Smile”: Cash Transfers and the Joys of Mothering in the Context of Poverty
Source: J Policy Anal Manage. Author manuscript; Available in PMC 2026 Jun 3. (PMC13229551; doi:10.1002/pam.70103)
Supplement: Appendix 1 — Table A: Stresses of motherhood (N=80). [file NIHMS2171028-supplement-Appendix_1.docx]

**Appendix**

Table A

*Stresses of motherhood (N=80)*

|  | n | Percent | Illustrative quote |
| --- | --- | --- | --- |
| Trying to get by on low-wage job or limited income | 31 | 39 | My lights actually was off and I was stressed out in the hospital wondering how I'm going to get my lights on because I just had a baby and I'm like, I need my lights on. |
| Responding to children’s needs | 30 | 38 | When she's screaming and hollering and I can't figure out why because it stresses me to, you know, figure it out. |
| Having to do everything yourself or not getting a break | 27 | 34 | The hard part is basically, I want to say, trying to do everything by yourself, trying to be the strong person that everybody thinks you are. |
| Managing daily family life | 26 | 33 | I guess trying to stay organized because it's a lot hard coming here and then at my mom's house and there’s different routines. And he just has so much stuff and it gets overwhelming because there's not that much space to put stuff. |
| Children’s misbehavior | 25 | 31 | She just like started like not listening all of a sudden soon as the baby came. She just started not listening. So I was aggravated a lot. |
| Mother’s own mental or physical health issues | 17 | 21 | It's really hard to work on my mental health, work on being mom, working on finding out who I truly am. |
| Worries about children’s health or development | 16 | 20 | She had the flu and she had croup. It closes the airway or throat close up and the breathing is hard. So that was very tough for me to see her like that. |
| Relationship issues with a romantic partner or child’s father | 14 | 18 | Right now I don't even have a co-parent to co-parent with. So, like her father had -- well her sperm donor has a girlfriend that tells him what he can and cannot do with his child. So yes, I just choose not to deal with him at all. |
| Transportation issues | 13 | 16 | Me not having a car would be like the most stressful thing. . . Getting on a bus with two kids and worrying about groceries with two kids and, you know, having twenty bags on the bus… |
| An unexpected pregnancy that was mistimed or unwanted | 13 | 16 | Oh my gosh, another baby. I don't know if I can afford to have it. … I was going to give birth no matter what, but I wasn't ready for him. |
| Worrying something will happen to children | 12 | 15 | The concern level, it go up, I guess, you know, you have another life you have to look over and make sure no harm comes to them. The concern level goes through the roof really. |
| Not having support people around | 10 | 13 | I mean I wish I had somebody to just be like, ‘Well okay I'm going to take [your daughter] for you’. Because its stressful. |
| Not having enough time with children | 7 | 9 | I really didn't want nobody to raise, like watch over my baby while I go to work. … Because I didn't want my baby to attach to nobody else but me. |
| Breastfeeding issues | 6 | 8 | I had to stop breastfeeding altogether, which broke my heart. |
| Work demands conflicting with family demands | 4 | 5 | I try to make sure that I don't neglect any parts of my home duties in order to be successful at work, but I also don't want to be unsuccessful at work because I'm still involved in all the things I have going on at home. |
| Housing issues | 4 | 5 | I need to get out of this house. I'm going crazy. … We need to move out of my mom's house. I need to go to work like we need to do just -- I guess time to just get the show on the road basically. |
| Child care issues | 3 | 4 | I work really early, from 6 to 6:30 and most of the daycares, they don't open until like 7. . . So, it was hard for me. |
| Worrying about a child's future | 3 | 4 | Just make sure he's prepared for the world, you know, around him. Society, and just everything, and I want him to know that he can be whatever he want to be, you know. |
| Children’s sleep issues | 2 | 3 | The least favorite parts is when she’s restless and she don’t want to go to sleep. . . And I’m tired. I want to go sleep. |
| Miscellaneous | 9 | 11 | You get that time and you feel like I'm not supposed to be doing this. You know, even if it's just to have a glass of wine or if it's just take a walk or you know anything. … I felt like I had to hurry up because I was just trying to get back home. And I was like, I'm supposed to be enjoying this time. |
| No stressors | 7 | 9 | I don't have any stressful things, thanks to God. … I'm enjoying with my kids. I'm so happy. |
|  | | | |
| *Source:* Authors’ tabulations and compilations. | | | |
